# Adipose‐Specific GHR Deletion Attenuates Brain Aging and Cognitive Decline in Aged Mice

**DOI:** 10.1111/acel.70407

**Published:** 2026-02-12

**Authors:** Yue Zhang, Rui Ma, Jia He, Liyuan Ran, Xuewei Yang, Yue Xi, Yingjie Wu

**Affiliations:** ^1^ Beijing Institute of Ophthalmology, Beijing Tongren Eye Center, Beijing Tongren Hospital, Capital Medical University, Beijing Ophthalmology & Visual Science Key Laboratory Beijing China; ^2^ Department of Physiology, College of Basic Medical Sciences, Liaoning Provincial Key Laboratory of Cerebral Diseases Dalian Medical University Dalian Liaoning China; ^3^ Institute for Genome Engineered Animal Models of Human Diseases, National Center of Genetically Engineered Animal Models for International Research, Liaoning Province Key Lab of Genetically Engineered Animal Models Department of Physiology, College of Basic Medical Sciences, Liaoning Provincial Key Laboratory of Cerebral Diseases Dalian Medical University Dalian Liaoning China; ^4^ Key Laboratory of Endocrine Glucose & Lipids Metabolism and Brain Aging, Ministry of Education Shandong Provincial Hospital Affiliated to Shandong First Medical University & Shandong Academy of Medical Sciences Jinan Shandong China; ^5^ Metabolism and Disease Research Center Central Hospital Affiliated to Shandong First Medical University, Shandong First Medical University & Shandong Academy of Medical Sciences Jinan Shandong China; ^6^ Department of Endocrinology and Metabolism The Third Affiliated Hospital of Jinzhou Medical University Jinzhou Liaoning China

**Keywords:** adipose tissue, brain aging, cognitive decline, GH/IGF‐1 axis, growth hormone receptor

## Abstract

Brain aging is marked by neuronal loss, synaptic deterioration, neuroinflammation, and proteinopathies, all of which contribute to cognitive decline. Although systemic suppression of growth hormone (GH)/insulin‐like growth factor‐1 (IGF‐1) signaling has been shown to extend lifespan and enhance cognitive function, the specific role of GH signaling in adipose tissue during brain aging remains unclear. In this study, we used aged (18–24 months) adipose‐specific GH receptor knockout (Ad‐GHRKO) mice and their littermate controls to investigate this relationship. We found that deletion of GHR in adipose tissue significantly attenuated key features of brain aging. Aged Ad‐GHRKO mice exhibited reduced neuronal loss in the cortex and hippocampus, diminished neuroinflammation, decreased cellular senescence, and lower levels of tau phosphorylation in the hippocampus compared to controls. Additionally, synaptic integrity, indicated by hippocampal PSD95 expression, and cortical neuronal excitability were preserved. Importantly, Ad‐GHRKO mice showed improved cognitive performance across multiple domains, including recognition memory (novel object recognition, NOR), working memory (Y‐maze), spatial learning/memory (Morris water maze), and associative fear memory (passive avoidance). These findings suggest adipose GH signaling as a previously unrecognized peripheral regulator of brain aging and cognition, implicating adipose GHR as a therapeutic target for mitigating age‐related cognitive decline.

## Graphical abstract

Adipose GHR deletion counteracts brain aging and cognitive decline in aged mice. This identifies adipose GH signaling as a peripheral regulator of neuroprotection and cognition.

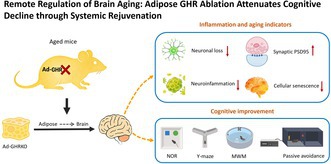

**Abbreviations**
aCSF: artificial cerebrospinal fluid
Ad‐GHRKO: adipose‐specific growth hormone receptor knockout
BSA: bovine serum albumin
DG: dentate gyrus
GH: growth hormone
GHR: growth hormone receptor
IGF‐1: insulin‐like growth factor‐1
IL: interleukin
MWM: Morris water maze
NOR: novel object recognition
PSD95: postsynaptic density protein 95
p‐Tau: phosphorylated tau
SASP: senescence‐associated secretory phenotype
SA‐β‐Gal: senescence‐associated β‐galactosidase
TNF‐α: tumor necrosis factor‐α

## 1 Introduction

Brain aging is a major risk factor for cognitive decline and neurodegenerative disorders. It is characterized by progressive neuronal loss, synaptic dysfunction, neuroinflammation, cellular senescence, and pathological protein aggregation (López‐Otín et al. 2023; Morrison and Baxter 2012). These changes collectively impair neuronal excitability and synaptic plasticity, ultimately compromising cognitive function (Navakkode and Kennedy 2024). Despite extensive research, the mechanisms underlying age‐related cognitive decline remain incompletely understood, and effective interventions to target brain aging are lacking. Identifying systemic modulators of brain aging and elucidating peripheral‐to‐central regulatory pathways are an urgent priority for developing targeted anti‐aging strategies.

The growth hormone (GH)/insulin‐like growth factor‐1 (IGF‐1) axis is a central neuroendocrine regulator of growth, metabolism, and aging, with increasingly recognized roles in brain function and cognition (Ashpole et al. 2015; Junnila et al. 2013). Both human and rodent studies show that circulating GH and IGF‐1 levels decline with age—a phenomenon termed the “somatopause” (Liu et al. 2007; Sonntag et al. 2005, 1980). Interestingly, genetic or pharmacological suppression of GH/IGF‐1 signaling extends lifespan and delays aging‐associated pathologies across species (Bartke 2008; Coschigano et al. 2003). Mice with global GH receptor (GHR) knockout (GHR^−/−^) or reduced GH signaling exhibit enhanced cognitive performance and resistance to age‐related memory decline (Kinney‐Forshee et al. 2004; Kinney et al. 2001). Similarly, transgenic mice expressing a GH receptor antagonist (GHA) show improved spatial learning and memory retention compared to wild‐type controls (Basu et al. 2017). These observations suggest that dampening GH/IGF‐1 signaling may exert neuroprotective effects during aging, though the contribution of specific tissues remains unresolved.

Adipose tissue has emerged as a metabolically active and endocrine organ that secretes a range of signaling molecules—including adipokines, cytokines, and growth factors—that influence both systemic physiology and brain function (Booth et al. 2016; Chen and Schneeberger 2024; Letra and Santana 2017; Scheja and Heeren 2019). Notably, adipose tissue expresses functional GHRs, and GH signaling in adipocytes modulates lipid metabolism, insulin sensitivity, and adipokine secretion (Berryman et al. 2016; Berryman and List 2017; Berryman et al. 2011). Previous studies have shown that adipose‐specific GHR knockout (Ad‐GHRKO) mice display increased adiposity, improved insulin sensitivity, and favorable metabolic profiles (List et al. 2019; List et al. 2022; Ran et al. 2019). However, whether adipose GH signaling contributes to brain aging and cognitive decline has not been examined. Given the central role of adipose tissue in systemic aging and the cognitive benefits associated with reduced GH signaling, we hypothesized that adipose‐specific GHR deletion would mitigate brain aging and preserve cognitive function in aged mice.

To test this, we used aged Ad‐GHRKO mice and conducted comprehensive histological, molecular, electrophysiological, and behavioral analyses. Our findings reveal that disruption of adipose GH signaling alleviates key features of brain aging—including neuronal loss, synaptic dysfunction, neuroinflammation, cellular senescence, and tau pathology—while preserving neuronal excitability and cognitive function. These results uncover a novel peripheral‐to‐central regulatory mechanism of brain aging and identify adipose GHR as a promising therapeutic target to counteract age‐related cognitive decline.

## 2 Materials and Methods

### 2.1 Materials

Sucrose, NaCl, KCl, K‐gluconate, MgCl_2_, MgSO_4_, CaCl_2_, NaH_2_PO_4_, NaHCO_3_, Mg‐ATP, Na‐GTP, HEPES, D‐glucose, paraformaldehyde, Triton X‐100, and 4′,6‐diamidino‐2‐phenylindole (DAPI) were purchased from Sigma‐Aldrich (St Louis, MO, USA). Non‐fat milk and bovine serum albumin (BSA) were obtained from Coolaber (Beijing, China). Additional materials and their sources are described in the relevant methodology sections.

### 2.2 Experimental Animals

Ad‐GHRKO mice were generated using a two‐step breeding strategy, as previously described (Ran et al. 2019). All mice were on a C57BL/6J genetic background. In the first step, GHR‐Floxed mice were intercrossed with Adipoq‐Cre mice to produce heterozygous offspring. These offspring were then intercrossed to obtain Ad‐GHRKO mice and their floxed littermate controls. Only male mice were used in this study. All animals were housed under standard conditions in an individually ventilated cage (IVC) system with a 12‐h light/dark cycle and were given ad libitum access to water and standard rodent chow (1010009, 12.79% kcal from fat; Xietong Bioengineering Company, China). Based on experimental requirements, mice were divided into two age groups: aged (18–24 months; corresponding to approximating 56–69 human years) and adult (5 months; equivalent to approximately 20–30 human years). All the animal experiments were approved by the Committee on the Ethics of Animal Experiments at Shandong Provincial Hospital, affiliated with Shandong First Medical University & Shandong Academy of Medical Sciences.

### 2.3 Behavioral Analysis

Behavioral assessments were conducted according to established protocols (Zhang et al. 2025).

#### 2.3.1 Novel Object Recognition (NOR)

Mice were habituated in a non‐glossy acrylic box for 5 min. During the acquisition phase, animals were allowed to explore two identical objects for 5 min. One of the objects was then replaced with a novel object, and exploration behavior was video‐recorded for an additional 5 min. The NOR index was calculated as:

$$\begin{array}{c} \mathrm{NOR}\;\text{Index}=\left(\text{Time spent exploring the novel object}\right)/\\ \;\left(\text{Total exploration time}\right). \end{array}$$

#### 2.3.2 Y‐Maze Spontaneous Alternation

Each mouse was placed at the center of a Y‐maze consisting of three symmetrical opaque arms and allowed to explore freely for 5 min. An arm entry was defined as all four limbs entering an arm. A spontaneous alternation was recorded when the mouse entered three different arms consecutively. The spontaneous alternation rate was calculated using the formula:

$$\text{Alternation Rate}=\left[\text{Number of alternations}/\left(\text{Total}\;\mathrm{arm}\;\text{entries}-2\right)\right].$$

#### 2.3.3 Morris Water Maze (MWM) Test

Spatial learning and memory were evaluated in a circular pool (120 cm diameter) filled with water maintained at 20°C ± 1°C. The pool was conceptually divided into four quadrants, with a transparent platform (10 cm diameter) submerged 1 cm below the surface in one quadrant. Visual cues were placed on the surrounding walls to assist spatial orientation. Training consisted of three trials per day (20 min inter‐trial interval) over 5 consecutive days. In each trial, mice were placed facing the pool wall at varying start points and given 90 s to locate the hidden platform. Mice that failed to find the platform were gently guided to it and allowed to stay for 10 s. Escape latency and swimming speed were recorded. 24 h after the final training session, a probe trial was conducted in which the platform was removed. Mice were allowed to swim freely for 90 s while the number of platform crossings and swim paths were recorded and analyzed.

#### 2.3.4 Passive Avoidance Test

A two‐compartment shuttle box with connected light and dark chambers separated by a guillotine door was used. During the acquisition phase, mice were placed in the illuminated compartment with free access to the dark side. Upon entry into the dark chamber, a mild foot shock (0.5 mA, 2 ms) was delivered. Mice remained in the apparatus for 5 min. 24 h later, the retention phase was conducted without any shock. The latency to enter the dark compartment and the total number of entries over a 5‐min observation period were recorded as measures of memory retention.

### 2.4 Slice Preparation and Whole‐Cell Patch‐Clamp Recording

Electrophysiological recordings were conducted as previously described (Wang et al. 2025). Mice were deeply anesthetized with isoflurane and rapidly decapitated. Brains were immediately extracted and placed in ice‐cold, oxygenated (95% O_2_/5% CO_2_) slicing solution containing (in mM): 175 sucrose, 2.5 KCl, 4 MgSO_4_, 0.5 CaCl_2_, 1.2 NaH_2_PO_4_, 26 NaHCO₃, and 25 D‐glucose (pH 7.3). Coronal forebrain slices (300 μm thick) were prepared using a vibrating microtome (Leica VT1200S, Wetzlar, Germany). Brain slices were allowed to recover for at least 1 h at room temperature (30°C–32°C) in oxygenated artificial cerebrospinal fluid (aCSF) containing: 124 mM NaCl, 2.5 mM KCl, 2 mM MgCl_2_, 2 mM CaCl_2_, 1.2 mM NaH_2_PO₄, 24 mM NaHCO₃, 5 mM HEPES, and 13 mM D‐glucose (pH 7.3). For recordings, individual slices were transferred to a submerged recording chamber and continuously perfused with oxygenated aCSF at a flow rate of 2 mL/min. Patch‐clamp recordings were obtained using glass pipettes (resistance: 3–5 MΩ) filled with an internal solution containing: 140 mM K‐gluconate, 7 mM NaCl, 4 mM Mg‐ATP, 0.3 mM Na‐GTP, and 10 mM HEPES (pH 7.3, 290–300 mOsm). Signals were recorded using an Axopatch 700B amplifier (Molecular Devices, USA), digitized at 10 kHz, and filtered between 0.1 and 5 kHz. Data acquisition and analysis were performed using pClamp 10.3 and Clampfit software (Molecular Devices).

### 2.5 Tissue Processing and Sectioning

Following deep anesthesia induced by intraperitoneal injection of 1% pentobarbital sodium (50 mg/kg), mice were transcardially perfused with ice‐cold 0.1 M phosphate buffer (PB, pH 7.4) followed by 4% paraformaldehyde in PB. Brains were carefully dissected and post‐fixed in the same fixative at 4°C for 24 h. Cryoprotection was performed by immersing the tissues sequentially in 15% sucrose in PB for 24 h and then in 30% sucrose in PB for 48–72 h at 4°C. Following cryoprotection, brains were embedded in OCT compound and rapidly frozen. Coronal sections (10 μm thickness) were prepared using a cryostat microtome.

### 2.6 SA‐β‐Gal Staining

Senescence‐associated β‐galactosidase (SA‐β‐Gal) activity was detected using a commercial staining kit (Beyotime, Cat# C0602, China) following the manufacturer's instructions. Brain sections were washed three times with PBS, fixed at room temperature for 40 min, and subsequently incubated overnight at 37°C in SA‐β‐Gal staining solution. Images were captured using a Pannoramic MIDI scanner (3DHISTECH Ltd., Budapest, Hungary).

### 2.7 Nissl Staining

Coronal brain sections from each experimental group were air‐dried at 37°C for 15 min. Sections were then washed three times in PBS (2 min each) with gentle agitation, followed by staining in 0.1% cresyl violet solution (Abcam, Cat# ab246816) for 4–6 min at room temperature. Excess stain was removed by three brief rinses (2 min each) in distilled water. Sections were dehydrated through a graded ethanol series (70%, 90%, 100%), cleared in xylene (2 × 3 min), and mounted using neutral resin.

### 2.8 Immunofluorescence Staining

Frozen brain sections were washed three times for 10 min each in PBS containing 0.3% Triton X‐100 (PBST) to permeabilize the tissue. Sections were then blocked for 1 h at room temperature in 2% BSA diluted in PBST. Primary antibodies diluted in blocking solution were applied overnight at 4°C: rabbit anti‐postsynaptic density protein 95 (PSD95, 1:100, Abcam Cat# ab18258) and mouse anti‐NeuN (1:200, Abcam Cat# ab104224). After three washes in PBST (10 min each), sections were incubated for 1 h at room temperature with the corresponding secondary antibodies: donkey anti‐mouse Alexa Fluor 594 (1:500, Invitrogen, Cat# A‐21203), goat anti‐rabbit Alexa Fluor 594 (1:500, Invitrogen, Cat# A‐11012), and DAPI for nuclear staining. Following three final PBST washes (10 min each), sections were mounted using anti‐fade media and imaged using a digital slide scanner (Pannoramic Digital Slide Scanners, 3DHISTECH, Budapest, Hungary).

### 2.9 Immunohistochemistry

On the first day, tissue processing followed the immunofluorescence protocol described above. On the second day, sections were washed three times with PBS and then incubated with biotin‐conjugated secondary antibodies for 2 h at room temperature. After secondary antibody incubation, the sections were washed three additional times with PBS and treated with avidin‐biotin complex solution (ABC Elite kit; Vector Laboratories, USA) for 30 min at room temperature. The signal was visualized by incubating sections with 3,3′‐diaminobenzidine (DAB, Sigma‐Aldrich, USA) until the desired staining intensity was reached, then the reaction was stopped by rinsing with PBS. Finally, sections were dehydrated through a graded ethanol series and coverslipped using neutral resin mounting medium.

### 2.10 Western Blot

Tissues were homogenized in 1**×** radioimmunoprecipitation assay (RIPA) lysis buffer supplemented with 1 mM phenylmethylsulfonyl fluoride (PMSF) and protease inhibitor cocktail. Lysates were centrifuged at 15,000**×** *g* for 15 min at 4°C, and supernatants were collected for further analysis. Protein concentrations were determined using a bicinchoninic acid (BCA) assay. Equal amounts of protein were loaded onto SDS‐PAGE gels for electrophoretic separation. Proteins were transferred to polyvinylidene difluoride (PVDF) membrane (Millipore, USA) via standard wet transfer. Membranes were blocked with 5% non‐fat milk in TBST for 1 h at room temperature, then incubated overnight at 4°C with primary antibodies diluted in blocking buffer: mouse anti‐PSD95 (1:1000; Abcam Cat# ab2723), rabbit anti‐IL‐10 (1:1000; Proteintech Cat# 82191‐3‐RR), rabbit anti‐IL‐6 (1:1000; CST Cat# 12912s), rabbit anti‐TNF‐α (1:1000; Proteintech Cat# 80258‐6‐RR), rabbit anti‐phosphor‐tau (S396) (1:1000; Abcam Cat# ab109390), and rabbit anti‐tau (1:1000; Abcam Cat# ab254256). Following three 10‐min washes in TBST, membranes were incubated with HRP‐conjugated secondary antibodies for 1 h at room temperature. After additional TBST washes (3 **×** 10 min), immunoreactive bands were visualized using enhanced chemiluminescence (ECL; Thermo Scientific, USA) and imaged with a Bio‐Rad ChemiDoc XRS (USA).

### 2.11 RNA Isolation and Quantitative Real‐Time PCR

RNA isolation and qPCR analysis were performed as previously described (Zhang et al. 2025). Total RNA was extracted from samples using Trizol Reagent (Takara Biotechnology, China). Subsequently, 1 μg of total RNA was reverse transcribed into cDNA using a First Strand cDNA Synthesis Kit (TransGen Biotech, China). Target gene expression levels were quantified by real‐time PCR on a CFX96 system (BIO‐RAD) using TransStart Top Green qPCR SuperMix (TransGen Biotech, China). Gene expression was calculated relative to the housekeeping gene GAPDH using the 2^−ΔΔCT^ method. Primer sequences are provided in Table 1.

**TABLE 1:** Primer list.

Primer	Forward primer 5′–3′	Reverse primer 5′–3′
GAPDH	CACTGGCATGGCCTTCCGT	CTTACTCCTTGGAGGCCAT
IL‐10	TCTGCCCTGTGAAAATAAGAGC	GTCAAACTCACTCATGGCTTTG
IL‐6	TCCATCCAGTTGCCTTCTTGG	CCACGATTTCCCAGAGAACATG
TNF‐α	CGTCAGCCGATTTGCTATCT	CGGACTCCGCAAAGTCTAAG

### 2.12 Data Analysis

For all morphometric analyses (including immunostaining, Nissl staining, and SA‐β‐Gal‐positive cell counting), specific regions of interest (ROIs) were defined according to anatomical landmarks and kept consistent across all samples. Areas of immunopositivity or cellular signals were measured within each ROI. The data were normalized by the corresponding ROI area to allow standardized comparison between samples. All quantifications were performed using ImageJ software.

Data are presented as mean ± SEM. For comparisons between two groups, unpaired two‐tailed Student's *t*‐tests or Mann–Whitney *U* tests were used, depending on data distribution. For comparisons involving multiple groups, one‐way or two‐way ANOVA was performed, followed by Tukey's post hoc test for multiple comparisons. Western blot band intensities were quantified with Image Lab software. Statistical analyses and graph generation were performed using GraphPad Prism version 9.0. Statistical significance was defined as *p* < 0.05 and is indicated as follows: *p < 0.05, **p < 0.01, ***p < 0.001, ****p < 0.0001.

## 3 Results

### 3.1 Adipose GHR Knockout Mitigates Neuronal Loss in Aging Mice

Neuronal loss is a hallmark of brain aging and a major contributor to cognitive decline in humans and rodent models alike (Burke and Barnes 2006; Morrison and Baxter 2012). This loss progressively affects vulnerable brain regions, including the cortex and hippocampus. To determine whether adipose‐specific GHR deletion alleviates age‐associated neuronal loss, we quantified neuronal density in these regions of aged mice.

**FIGURE 1:**
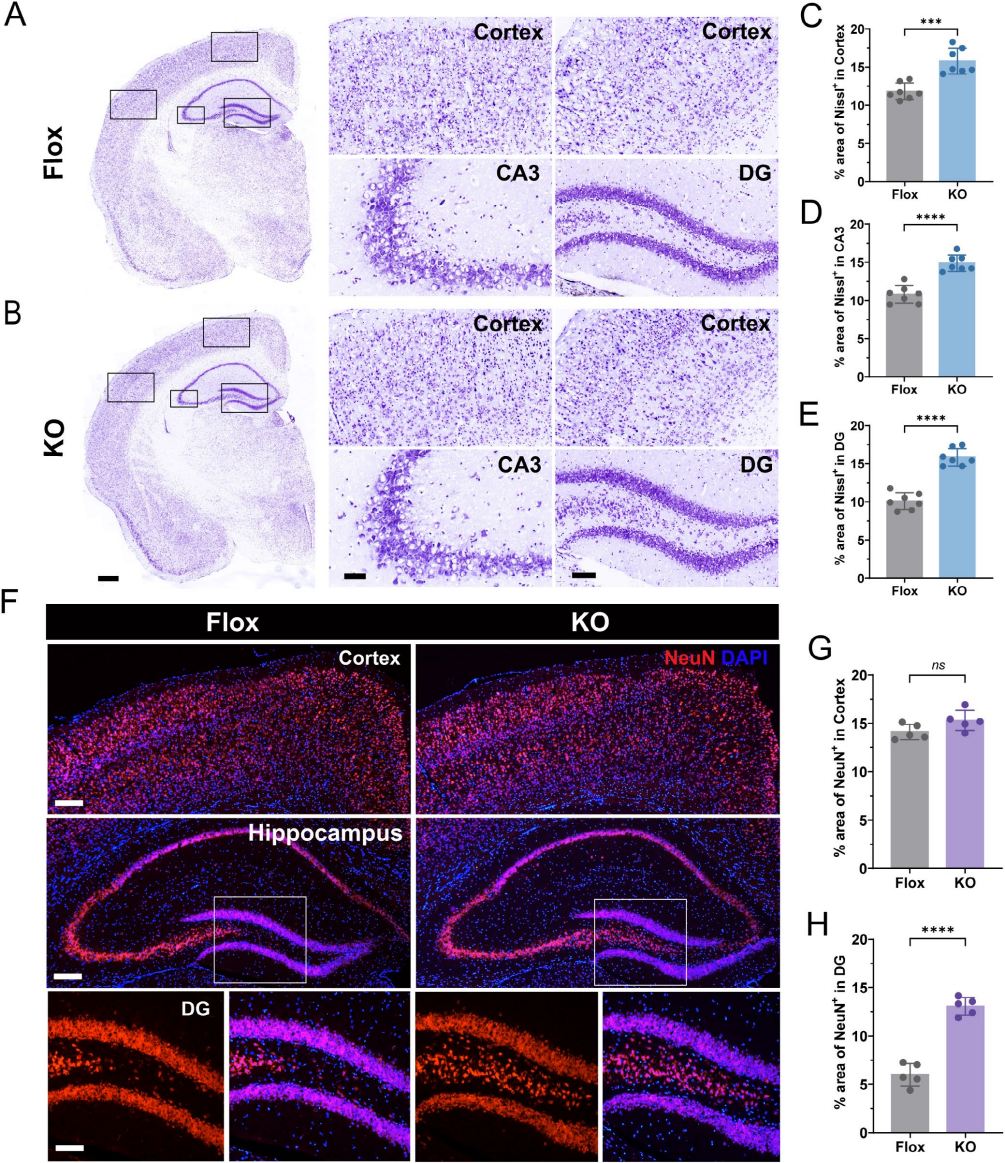
Adipose GHR knockout attenuates age‐related neuronal loss in aged mouse brains. (A, B) Representative photomicrographs of Nissl‐stained brain sections from aged Flox control (A) and Ad‐GHRKO (B) mice, showing the cortex, hippocampal CA3, and dentate gyrus (DG). Insets display higher magnification views of the boxed areas. Scale bars: 500 μm (whole brain, left), 100 μm (cortex and DG), 50 μm (CA3). (C–E) Quantification of Nissl‐positive neurons in the cortex (C), CA3 (D), and DG (E) regions (*n* = 7). (F) Representative immunofluorescence images of NeuN‐positive neurons (red) in the cortex and hippocampus of aged Flox and Ad‐GHRKO mice. Nuclei are counterstained with DAPI (blue). Scale bars: 200 μm (cortex and hippocampus), 100 μm (DG). (G, H) Quantification of NeuN‐positive cells in the cortex (G) and DG (H) (*n* = 5). Data are presented as mean ± SEM; ***p < 0.001, ****p < 0.0001; ns, not significant.

Representative Nissl staining revealed notable morphological differences between aged control (Flox) and Ad‐GHRKO mice (Figure 1A,B). Quantitative analysis showed a significant increase in Nissl‐positive neurons in the aged Ad‐GHRKO group compared to the Flox control across all examined areas: cortex (Figure 1C), hippocampal CA3 (Figure 1D), and dentate gyrus (DG) (Figure 1E). To further confirm the neuroprotective effect, we performed NeuN immunofluorescence staining. While an increasing trend was observed in the cortex (Figure 1F,G), a statistically significant increase in NeuN‐positive cells was specifically detected in the DG of aged Ad‐GHRKO mice compared to controls (Figure 1F,H). Together, these data, particularly the consistent and robust effects observed in the hippocampus, indicate that adipose‐specific GHR deletion effectively attenuates neuronal loss during aging in key brain regions.

### 3.2 Adipose GHR Deletion Enhances Hippocampal Synaptic Markers and Attenuates Neuroinflammation in Aged Mice

**FIGURE 2:**
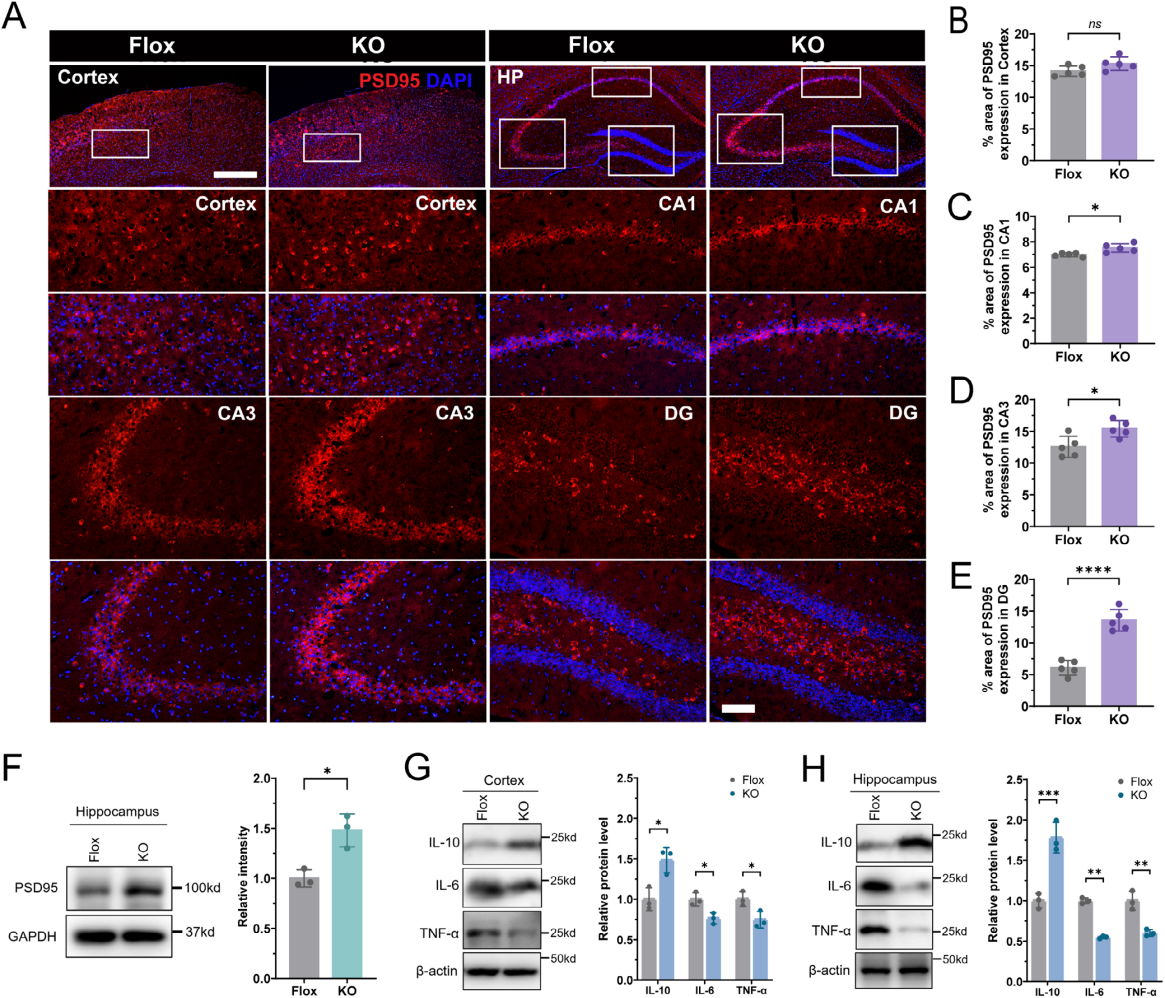
Adipose GHR deletion enhances hippocampal synaptic markers and attenuates neuroinflammation in aged mice. (A) Representative immunofluorescence images showing PSD95 (red) expression in the cortex and hippocampus of aged Flox control and Ad‐GHRKO mice. Nuclei are counterstained with DAPI (blue). Scale bar: 500 μm. Insets display higher‐magnification views of boxed regions in the cortex, hippocampal CA1, CA3, and dentate gyrus (DG). Scale bars: 100 μm. (B–E) Quantification of PSD95 fluorescence intensity in the cortex (B), CA1 (C), CA3 (D), and DG (E) (*n* = 5). (F) Western blot analysis of PSD95 protein levels in hippocampal lysates from aged mice (*n* = 3). (G, H) Western blot analysis of IL‐10, IL‐6, and TNF‐α protein levels in the cortex (G) and hippocampus (H) of aged mice (*n* = 3). Data are presented as mean ± SEM; *p < 0.05, **p < 0.01, ***p < 0.001, ****p < 0.0001; ns, not significant.

Synaptic plasticity underlies learning and memory processes, and alterations in synaptic protein expression—particularly within the hippocampus—are hallmark features of brain aging that closely correlate with cognitive decline (Navakkode and Kennedy 2024) (Hara et al. 2014; Masliah et al. 1993). Postsynaptic density protein 95 (PSD95) is a crucial scaffolding protein that maintains postsynaptic structure and function (Migaud et al. 1998; Zeng et al. 2018). To assess synaptic integrity following adipose‐specific GHR deletion, we examined PSD95 expression in aged mice. Immunofluorescence analysis revealed region‐specific increases in PSD95 in aged Ad‐GHRKO mice (Figure 2A). While PSD95 fluorescence intensity in the cortex was comparable between Ad‐GHRKO and Flox controls (Figure 2B), significant elevations were observed in hippocampal CA1 (Figure 2C), CA3 (Figure 2D), and DG subregions (Figure 2E). Western blot analysis of whole hippocampal lysates confirmed increased PSD95 protein levels in aged Ad‐GHRKO mice relative to controls (Figure 2F, Figure S1A,B).

Neuroinflammation, a hallmark of aging (“neuroinflammaging”), contributes to neuronal dysfunction and synaptic vulnerability (Franceschi et al. 2018; Norden et al. 2015), directly impairing plasticity and cognition (Norden and Godbout 2013; Patterson 2015). To determine whether adipose GHR deletion modulates this inflammatory milieu, we measured key cytokines at the protein level in both cortex and hippocampus. Western blot analysis revealed that aged Ad‐GHRKO mice exhibited a significant anti‐inflammatory shift in both regions, with reduced pro‐inflammatory cytokines (IL‐6, TNF‐α) and increased anti‐inflammatory cytokine IL‐10 (Figure 2G,H, Figure S3A). This protein‐level profile is consistent with mRNA expression patterns (Figure S2A,B). In contrast, young (5‐month‐old) Ad‐GHRKO mice showed no significant differences in IL‐10, IL‐6, or TNF‐α levels compared to age‐matched controls (Figures S2C,D and S3B), indicating that the attenuated neuroinflammatory phenotype is acquired with aging.

Together, these data demonstrate that adipose‐specific GHR deletion enhances hippocampal synaptic protein expression and mitigates age‐related neuroinflammation.

### 3.3 Adipose GHR Deletion Attenuates Cellular Senescence in the Aged Brain

**FIGURE 3:**
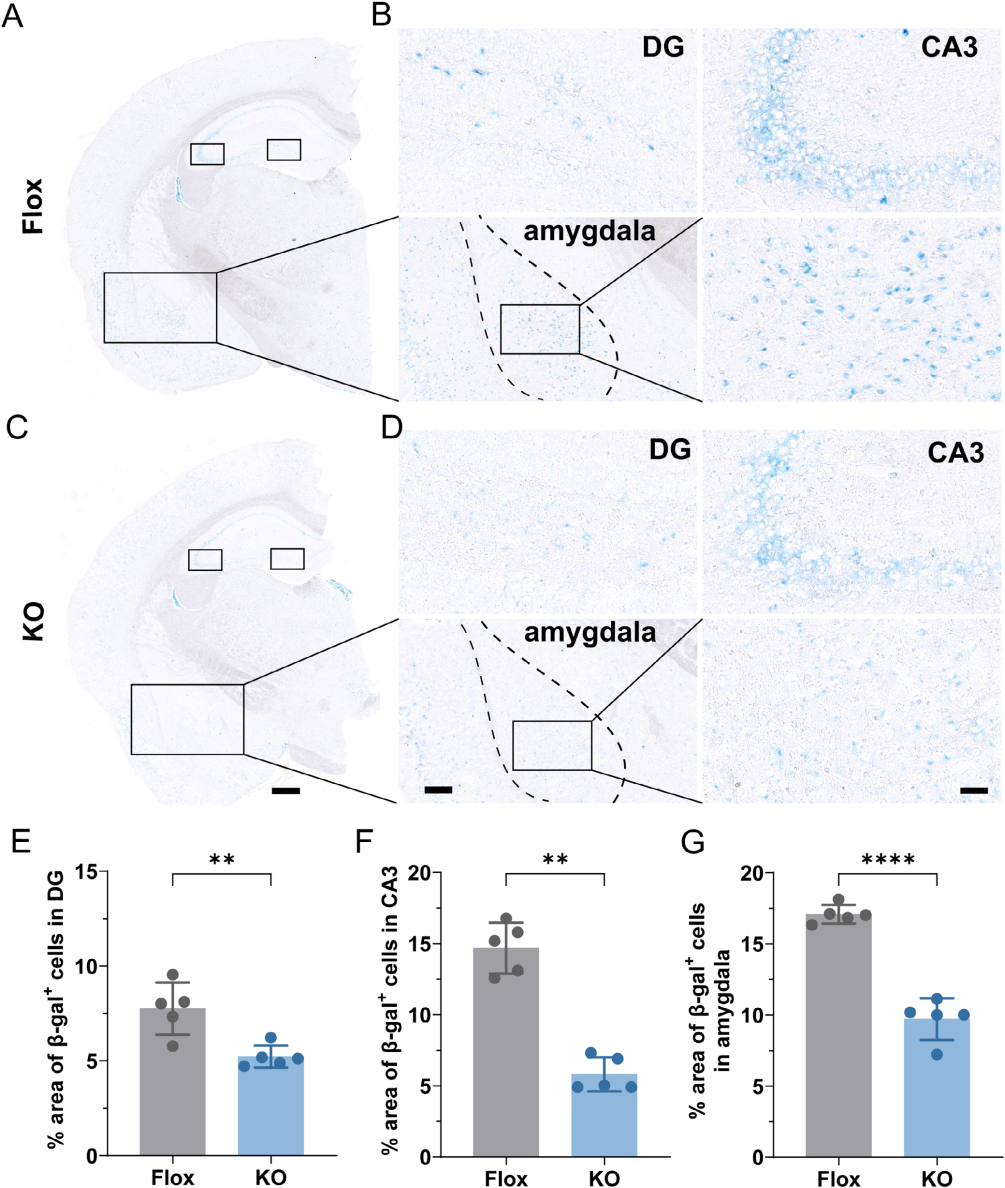
Adipose GHR deletion reduces cellular senescence in aged mouse brains. (A, C) Representative low‐magnification images of SA‐β‐Gal‐stained brain sections from aged Flox control (A) and Ad‐GHRKO (C) mice, scale bars: 500 μm. (B, D) High‐magnification images of SA‐β‐Gal‐positive cells (blue) in the hippocampal dentate gyrus (DG), CA3, and amygdala in Flox (B) and Ad‐GHRKO (D) mice, scale bars: 200 μm. Insets display higher‐magnification views of boxed areas, scale bars: 50 μm. (E–G) Quantification of SA‐β‐Gal‐positive cell density in the DG (E), CA3 (F), and amygdala (G) of aged mice (*n* = 5). Data are presented as mean ± SEM; **p < 0.01, ****p < 0.0001.

The accumulation of senescent cells—characterized by irreversible cell cycle arrest and a pro‐inflammatory senescence‐associated secretory phenotype (SASP)—is a key driver of tissue dysfunction and a hallmark of aging (Childs et al. 2015; López‐Otín et al. 2013). In the central nervous system, senescent neurons and glial cells contribute to neuroinflammation, synaptic impairment, and cognitive decline during aging (Bussian et al. 2018; Musi et al. 2018; Thapa et al. 2024). SA‐β‐Gal activity is widely used as a biomarker to identify senescent cells in aged tissues. To assess the effects of adipose‐specific GHR deletion on brain senescence, we performed SA‐β‐Gal staining on brain sections from aged mice. Low‐magnification whole‐brain images showed senescent cell distribution in aged Flox controls (Figure 3A) and Ad‐GHRKO mice (Figure 3C). High‐magnification analysis focused on senescence‐prone regions including the hippocampal DG, CA3, and amygdala (Figure 3B,D). Ad‐GHRKO mice exhibited markedly fewer SA‐β‐Gal‐positive (blue staining) senescent cells compared to Flox controls across all regions examined. Quantitative analysis confirmed a significant reduction in SA‐β‐Gal‐positive cell density in the DG (Figure 3E), CA3 (Figure 3F), and amygdala (Figure 3G) of Ad‐GHRKO mice. These findings demonstrate that adipose‐specific GHR deletion effectively attenuates cellular senescence in key brain regions involved in cognitive and emotional functions during aging.

### 3.4 Adipose GHR Deletion Reduces Age‐Associated Tau Phosphorylation in the Hippocampus of Aged Mice

Pathological tau hyperphosphorylation is a hallmark of Alzheimer's disease, but accumulating evidence shows that phosphorylated tau (p‐Tau) is also increased during normal brain aging, independently of neurofibrillary tangle formation, contributing to synaptic dysfunction and cognitive decline (Duff et al. 2000; Dujardin et al. 2020). Soluble p‐Tau species disrupt synaptic vesicle trafficking, impair mitochondrial function, and sensitize neurons to degeneration. Given its role in age‐related neuronal vulnerability, we investigated whether adipose‐specific GHR deletion affects p‐Tau accumulation in aged brain.

**FIGURE 4:**
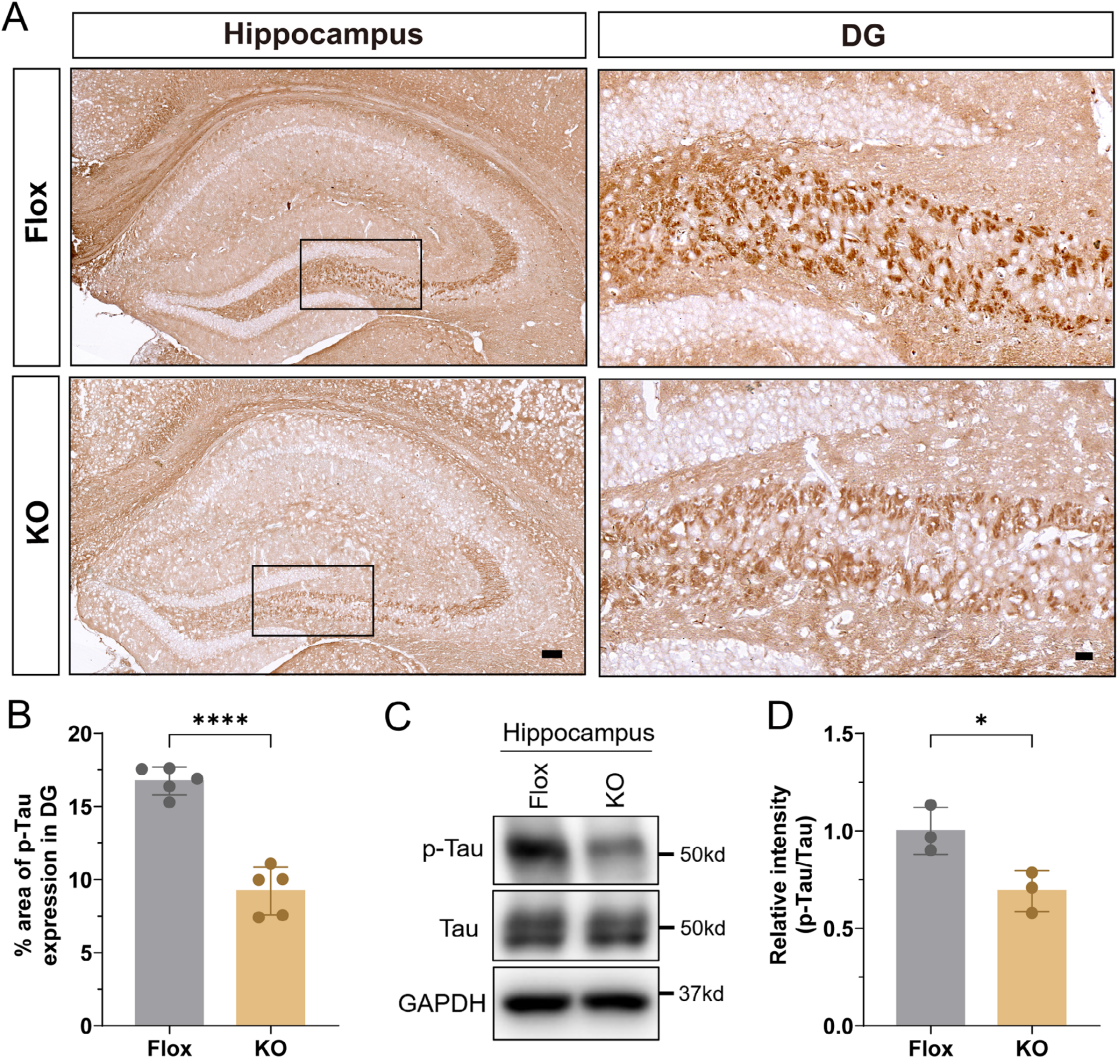
Adipose GHR deletion reduces tau phosphorylation in the aged dentate gyrus. (A) Representative immunohistochemistry images of phosphorylated tau (p‐Tau) in the hippocampal dentate gyrus (DG) of aged Flox control and Ad‐GHRKO mice. Insets show higher‐magnification views of the DG subregion. Scale bars: 100 μm (main images), 20 μm (insets). (B) Quantification of p‐Tau immunoreactivity in the DG (*n* = 5). (C, D) Western blot analysis of hippocampal p‐Tau. (C) Representative blots of p‐Tau and total Tau. (D) Quantification of the p‐Tau/Tau ratio (*n* = 3). Data are presented as mean ± SEM; *p < 0.05, ****p < 0.0001.

Immunohistochemical analysis revealed markedly reduced p‐Tau immunoreactivity in the DG of aged Ad‐GHRKO mice compared to Flox controls (Figure 4A). Quantification confirmed a significant decrease in DG‐specific p‐Tau intensity (Figure 4B). Consistent with these results, western blot analysis of hippocampal lysates showed a significantly lower ratio of p‐Tau to total tau protein in Ad‐GHRKO mice relative to controls (Figure 4C,D, Figure S1C–E). These findings indicate that adipose GHR deletion attenuates age‐associated tau phosphorylation in the hippocampus, suggesting a novel peripheral metabolic mechanism that reduces neuronal vulnerability during brain aging.

### 3.5 Adipose GHR Deletion Preserves Neuronal Excitability in Aged Cortex

**FIGURE 5:**
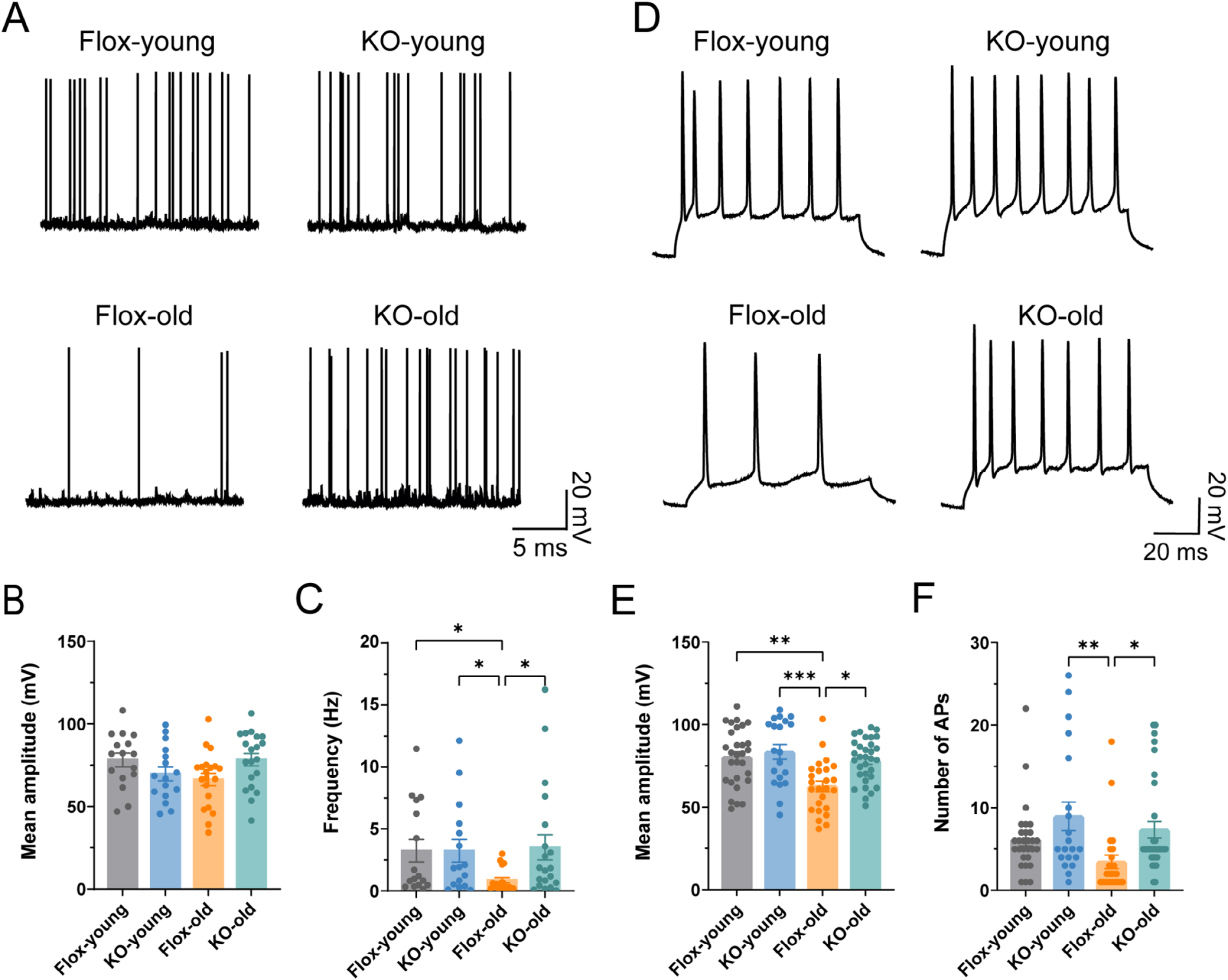
Adipose GHR deletion rescues age‐related deficits in cortical neuronal excitability. (A) Representative traces of spontaneous action potentials recorded from cortical neurons in young (5 months) and aged (18–24 months) Flox control and Ad‐GHRKO mice. (B, C) Quantification of spontaneous action potential amplitude (B) and frequency (C). Sample sizes: Flox‐young (*n* = 16 cells), KO‐young (*n* = 16 cells), Flox‐old (*n* = 20 cells), KO‐old (*n* = 20 cells). (D) Representative traces of evoked action potentials in response to current injection. (E, F) Quantification of evoked action potential amplitude (E) and number of spikes per stimulus (F). Sample sizes: Flox‐young (*n* = 29 cells), KO‐young (*n* = 20 cells), Flox‐old (*n* = 25 cells), KO‐old (*n* = 32 cells). Data are presented as mean ± SEM; *p < 0.05, **p < 0.01, ***p < 0.001 (two‐way ANOVA with Tukey's post hoc test). Group abbreviations: Flox‐young, young control; KO‐young, young Ad‐GHRKO; Flox‐old, aged control; KO‐old, aged Ad‐GHRKO.

Neuronal excitability and action potential fidelity are critical for neural circuit function, and age‐related changes in intrinsic membrane properties contribute significantly to cognitive decline during normal brain aging (Disterhoft and Oh 2007). To investigate whether adipose‐specific GHR deletion preserves neuronal excitability, we performed whole‐cell patch‐clamp recordings on cortical neurons from young (5 months) and aged (18–24 months) Flox control and Ad‐GHRKO mice. Analysis of spontaneous action potentials revealed age‐dependent changes (Figure 5A–C). While action potential amplitude remained stable across groups (Figure 5B), spontaneous firing frequency showed a significant age **×** genotype interaction (Figure 5C). Aged Flox mice exhibited markedly reduced firing frequency compared to young controls, whereas neurons of aged Ad‐GHRKO mice maintained firing frequencies comparable to young mice, indicating a full rescue of age‐related spontaneous activity deficits.

Evoked action potential recordings showed similar trends (Figure 5D–F). Although no genotype differences were observed in young mice, neurons of aged Flox mice exhibited significantly reduced action potential amplitude (Figure 5E) and fewer evoked spikes per stimulus (Figure 5F). Adipose‐specific GHR deletion prevented these deficits, preserving both amplitude and spike number at youthful levels in aged mice. These electrophysiological data demonstrate that adipose GHR deletion maintains essential biophysical properties of cortical neurons, preserving spontaneous and evoked firing during aging.

### 3.6 Adipose GHR Deletion Rescues Cognitive Deficits in Aged Mice

**FIGURE 6:**
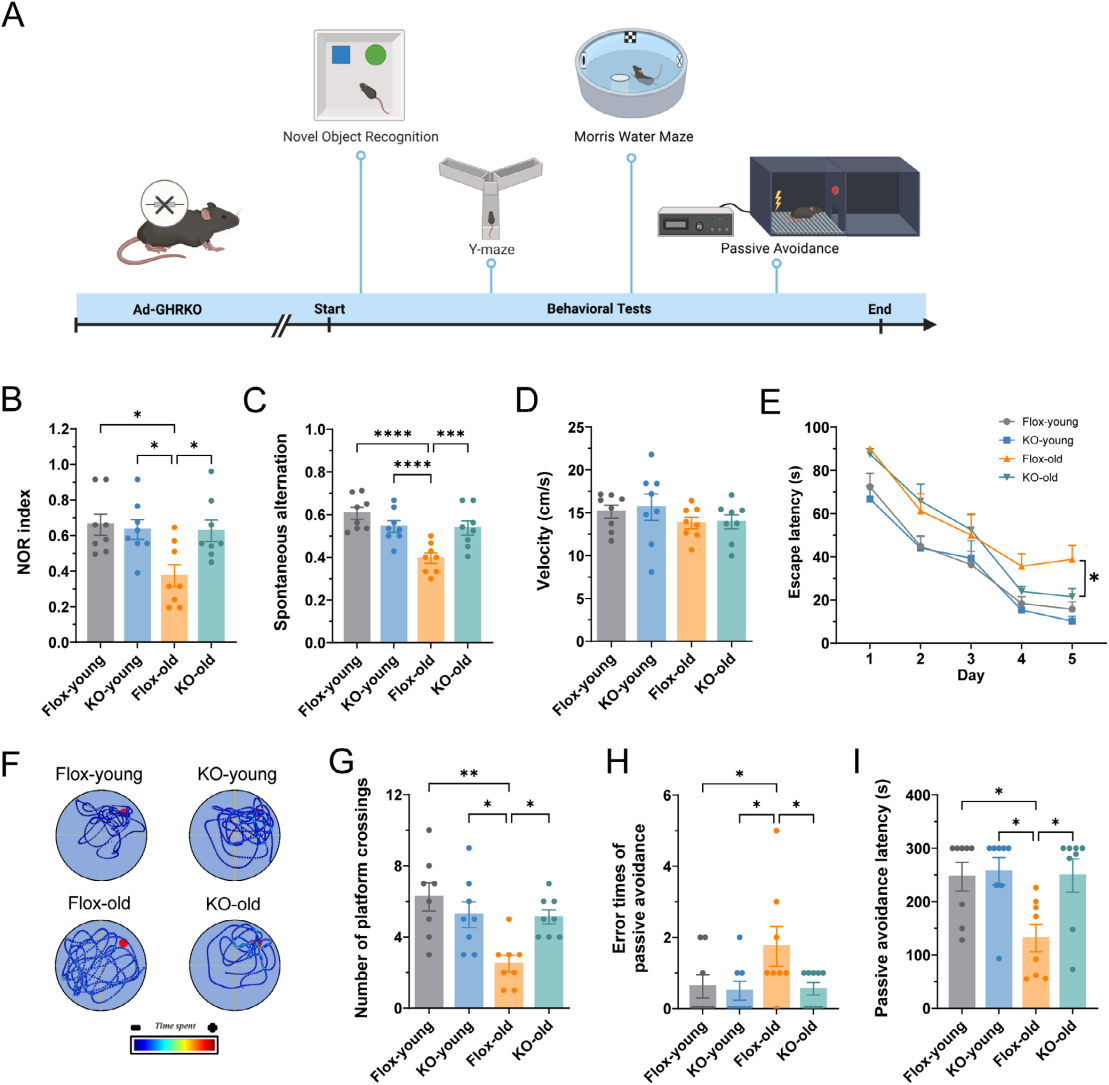
Adipose GHR deletion ameliorates cognitive decline in aged mice. (A) Schematic of the behavioral testing timeline. (B) Novel Object Recognition (NOR) index in young and aged mice. (C) Spontaneous alternation rate in the Y‐maze test. (D–G) Morris water maze test: (D) Swimming velocity, (E) Escape latency during acquisition training, (F) Representative swim paths during the probe test, (G) Number of platform crossings during the probe test. (H–I) Passive avoidance test: (H) Number of errors (entries into the dark compartment), (I) Latency to enter the dark compartment. Data are presented as mean ± SEM; *n* = 8 per group; *p < 0.05, **p < 0.01, ***p < 0.001, ****p < 0.0001 (two‐way ANOVA with Tukey's post hoc test). Group abbreviations: Flox‐young, young control; KO‐young, young Ad‐GHRKO; Flox‐old, aged control; KO‐old, aged Ad‐GHRKO.

To assess the effects of adipose‐specific GHR knockout on cognitive aging, young and aged Flox control and Ad‐GHRKO mice underwent a battery of behavioral tests (Figure 6A). In the NOR test, which evaluates recognition memory based on novelty preference, young Flox and Ad‐GHRKO mice performed similarly. However, aged Flox mice showed a significant decline in NOR index compared to young controls (Figure 6B). This deficit was reversed in aged Ad‐GHRKO mice, indicating that adipose GHR deletion attenuates age‐related recognition memory impairment. In the Y‐maze test, assessing working memory, no differences were observed between genotypes in young mice, but aged Flox mice displayed significantly reduced spontaneous alternation rates (Figure 6C). This deficit was ameliorated in aged Ad‐GHRKO mice, demonstrating preserved working memory capacity. Morris water maze testing confirmed comparable swimming speeds across all groups (Figure 6D), ruling out motor impairments. Aged Flox mice exhibited prolonged escape latencies during training sessions (Figure 6E) and fewer platform crossings in probe trials (Figure 6F,G), indicative of spatial memory deficits. In contrast, aged Ad‐GHRKO mice showed significantly better performance, with shorter escape latencies and increased platform crossings, confirming rescue of spatial learning and memory (Figure 6E–G).

Finally, in the passive avoidance test, which measures associative fear memory, young mice showed no genotype differences. Aged Flox mice exhibited increased errors and decreased latency to enter the dark compartment, both of which were significantly attenuated in aged Ad‐GHRKO mice (Figure 6H,I), reflecting preserved aversive learning. Collectively, these results demonstrate that adipose‐specific GHR deletion effectively mitigates age‐related cognitive decline across multiple domains.

## 4 Discussion

This study provides evidence that adipose tissue acts as a key peripheral regulator of brain aging, as demonstrated by the effects of its targeted GHR deletion. We demonstrate that adipose‐specific GHR knockout attenuates multiple hallmarks of brain aging—including neuronal loss, synaptic dysfunction, neuroinflammation, cellular senescence, and tau pathology—ultimately preserving neuronal excitability and cognitive function in aged mice. These findings suggest a novel endocrine pathway through which adipose GH signaling influences brain aging, offering a tissue‐specific therapeutic target against age‐related cognitive decline.

### 4.1 Adipose‐Brain Crosstalk in Neuroinflammaging and Synaptic Integrity

Our data indicate that adipose GHR ablation shifts the brain's cytokine milieu toward an anti‐inflammatory state, characterized by decreased IL‐6 and TNF‐α and increased IL‐10 (Figure 2G,H). This immunomodulation likely underlies the observed synaptic preservation, particularly the enhanced PSD95 expression in hippocampal subfields (Figure 2A–F). Adipose tissue secretes a diverse array of immunoregulatory adipokines, such as adiponectin and leptin, whose production is modulated by GH signaling (Fain et al. 1999; Kopchick et al. 2020). Adiponectin promotes hippocampal neurogenesis via AdipoR1/AMPK pathways (Yau et al. 2014), while leptin regulates synaptic plasticity through NMDA receptor trafficking (McGregor and Harvey 2019). The reduction in neuroinflammation aligns with previous studies demonstrating that systemic GH suppression decreases CNS inflammatory mediators (Velloso and Donato 2024; Young et al. 2020). Elevated IL‐10 may directly inhibit microglial SASP, as IL‐10 deficiency exacerbates age‐related microglial activation and cognitive decline (Norden and Godbout 2013). Thus, adipose‐derived anti‐inflammatory signals may foster a permissive microenvironment for synaptic maintenance.

### 4.2 Peripheral Control of Brain Senescence and Proteostasis

The significant reduction of SA‐β‐Gal‐positive cells in Ad‐GHRKO brains provides the first evidence that adipose‐specific intervention can mitigate cellular senescence in the brain. This decrease was prominent in the DG, a region where senescent cell accumulation drives neurogenic decline and tau pathology (Ben Abdallah et al. 2010). Senescent glia promote neurodegeneration through SASP factors that disrupt blood–brain barrier integrity and enhance tau hyperphosphorylation (Bussian et al. 2018). The observed reduction in p‐Tau (S396) specifically within the DG supports this connection, as soluble p‐Tau oligomers impair mitochondrial trafficking and synaptic function independent of neurofibrillary tangles (Lasagna‐Reeves et al. 2011). The DG's vulnerability to age‐related damage likely explains why adipose GHR deletion exerts maximal protective effects here. Importantly, reduced cellular senescence within the DG niche may rejuvenate the neurogenic microenvironment (Bussian et al. 2018).

### 4.3 Restoration of Neuronal Excitability and Cognitive Resilience

Electrophysiological recovery in the Ad‐GHRKO cortex, evidenced by restored spontaneous firing frequency and evoked spike generation, provides a functional substrate for preserved cognition. Age‐related declines in neuronal excitability are primarily driven by potassium channel dysregulation and calcium homeostasis disruption (Thibault and Landfield 1996; Urrutia et al. 2024), processes modulated by inflammatory cytokines whose levels were reduced in aged Ad‐GHRKO brains. Although circulating IGF‐1 likely decreases with GHR loss, attenuated neuroinflammation and improved insulin sensitivity (List et al. 2022) may compensate for central IGF‐1 alterations. Critically, adipose‐specific targeting achieves broad cognitive rescue—including recognition, spatial, and fear memory—while avoiding the systemic developmental defects (e.g., growth impairment, skeletal immaturity) associated with global GH/IGF‐1 suppression (Lupu et al. 2001; Yakar and Isaksson 2016). This approach effectively counters brain aging and offers a safer therapeutic strategy for age‐related cognitive decline.

### 4.4 The DG's Role in Cognitive Longevity

Our findings align with prior studies in long‐lived Ames dwarf mice, where GH/IGF‐1 deficiency enhances DG neurogenesis and preserves cognition during aging (Sun et al. 2005). Despite systemic IGF‐1 suppression, Ames dwarfs maintain elevated hippocampal IGF‐1 and increased BrdU^+^/NeuN^+^ neurons in the DG—mirroring the DG‐focused protection observed in Ad‐GHRKO mice. This convergence suggests that: (i) DG neurogenesis is a conserved mechanism for cognitive longevity in GH‐signaling‐modified models, and (ii) adipose‐derived signals may amplify endogenous DG repair programs. However, the potential contribution of enhanced neurogenesis in our Ad‐GHRKO mice, while a compelling hypothesis consistent with the observed DG preservation, has not been directly assessed and represents an important direction for future research.

### 4.5 Therapeutic Implications and Limitations

Our findings suggest that adipose GHR is a promising therapeutic target for healthy brain aging. Compared to CNS‐directed interventions, adipose modulation offers greater translational feasibility. However, several limitations of our study must be acknowledged. First, our study was conducted exclusively in male mice to maintain direct comparability with the foundational metabolic characterization of the Ad‐GHRKO model, which was performed in males (Ran et al. 2019). As a result, our findings do not address potential sex‐dependent effects of adipose GHR signaling on brain aging, an important area for future investigation. Second, although the Adipoq‐Cre driver provides high adipose specificity and our validation showed no detectable GHR recombination in whole brain (Ran et al. 2019), we cannot fully exclude the theoretical possibility of very low‐level, cell‐type‐specific Cre activity. Nevertheless, our genetic data indicate that any potential leakiness is insufficient to produce a detectable knockout event in the brain and is therefore unlikely to drive the observed neuroprotective phenotypes.

### 4.6 Future Perspectives

The protective effects observed in Ad‐GHRKO mice likely involve multiple, interconnected mechanisms. Beyond the local attenuation of neuroinflammation and cellular senescence documented here, we propose that systemic metabolic remodeling of adipose tissue represents a key upstream event. The improved metabolic profile of these mice, including enhanced insulin sensitivity and the distinctive “healthy obese” phenotype with increased subcutaneous lipid storage and buffering capacity (Ran et al. 2019), likely mitigates chronic peripheral metabolic stress such as glycemic instability and lipotoxicity. We speculate that this reduction in systemic drivers of aging creates a permissive environment that indirectly preserves brain function. Future studies are needed to define the hierarchy of events and identify principal mediators. Critical questions include: (1) whether the neuroprotection arises from specific adipose‐derived factors or overall systemic metabolic improvement; and (2) how adipose GHR deletion affects local IGF‐1 signaling within the DG niche.

## 5 Conclusion

Our findings demonstrate that adipose GH signaling is a potent modulator of brain aging through multi‐level neuroprotection, with the DG emerging as a key target. By showing that peripheral metabolic manipulation can ameliorate fundamental aging processes in the DG—including cellular senescence, tau phosphorylation, and synaptic dysfunction—this work provides a foundation for a new paradigm to combat age‐related cognitive decline. Future research should aim to identify adipose‐derived factors that selectively enhance DG neurogenesis and explore translational strategies for adipose GHR modulation.

## Author Contributions

Y.Z. designed the experiments, performed experiments and data analysis, prepared the figures, and wrote and revised the manuscript. J.H. performed experiments and analyzed data. R.M. and L.R. provided the mice. X.Y. performed electrophysiological experiments. Y.W. designed experiments and revised the manuscript. Y.X. revised the manuscript. Y.Z. and Y.W. provided financial support. All authors read and approved the final manuscript.

## Funding

This work was supported by National Key Research and Development Program of China, 2022YFE0132200, 2021YFA0805100, 2021YFF0702100. National Natural Science Foundation of China, 82101275, 82370866.

## Conflicts of Interest

The authors declare no conflicts of interest.

## Supporting information


**Data S1:** acel70407‐sup‐0001‐DataS1.pdf.

## Data Availability

Data are available upon request to the corresponding author.
