# Supplementary material for: Adipose‐Specific GHR Deletion Attenuates Brain Aging and Cognitive Decline in Aged Mice
Source: Aging Cell. 2026 Feb 12;25(2):e70407. doi: 10.1111/acel.70407 (PMC12900898; doi:10.1111/acel.70407)
Supplement: Supplementary file 1 — Data S1: acel70407‐sup‐0001‐DataS1.pdf. [file ACEL-25-e70407-s001.pdf]

Adipose-Specific GHR Deletion Attenuates Brain Aging and Cognitive Decline in Aged Mice

Supplementary Figures

Supplementary Figure S1

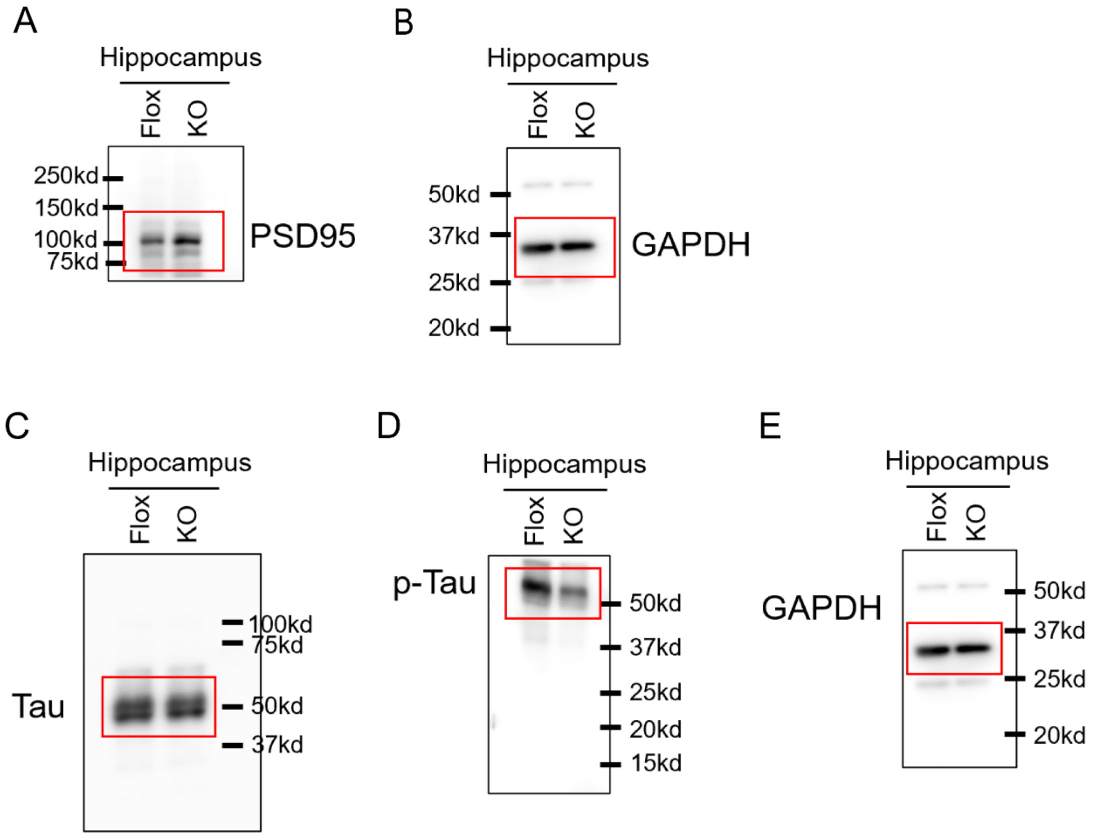

Supplementary Figure S1. Full-length, uncropped western blot images.

(A, B) Original, uncropped blots for PSD95 (A) and the corresponding loading control GAPDH (B) shown in Figure 2F.

(C-E) Original, uncropped blots for total Tau (C), phosphorylated Tau (p-Tau, D), and the corresponding loading control GAPDH (E) associated with analyses presented in Figure 4C.

Molecular weight markers (kd) are indicated. Relevant lanes used for final figures are outlined.

Supplementary Figure S2

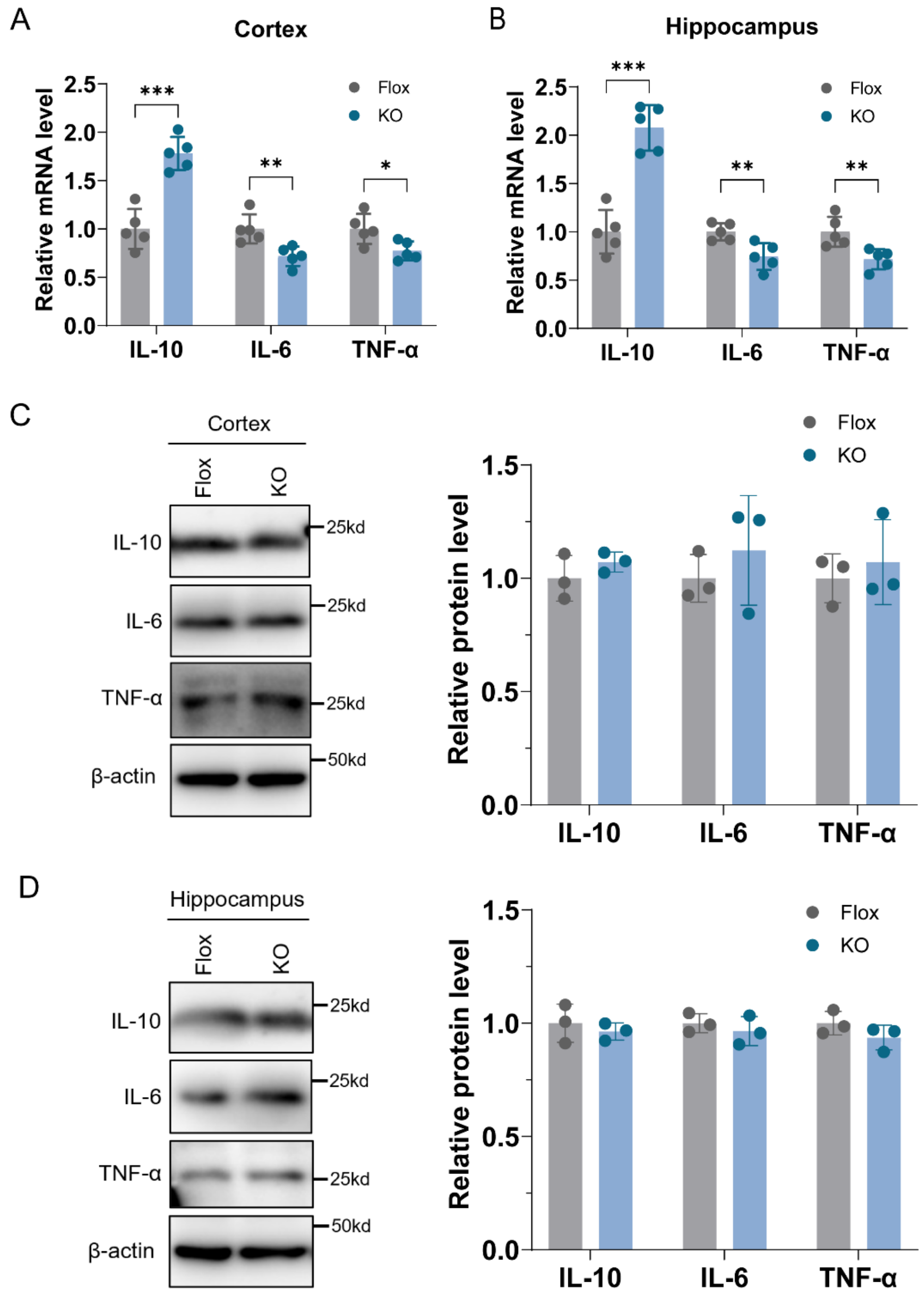

Supplementary Figure S2. Cytokine expression analyses in the cortex and hippocampus.

(A, B) qPCR analysis of IL-10, IL-6 and TNF-α mRNA levels in the cortex (A) and hippocampus (B) of aged Flox control and Ad-GHRKO mice (n = 5).

(C, D) Western blot analysis of IL-10, IL-6 and TNF-α protein levels in the cortex (C) and hippocampus (D) of young (5-month-old) Flox control and Ad-GHRKO mice (n = 3).

Data are presented as mean ± SEM. *$P < 0.05$, **$P < 0.01$, ***$P < 0.001$.

Supplementary Figure S3

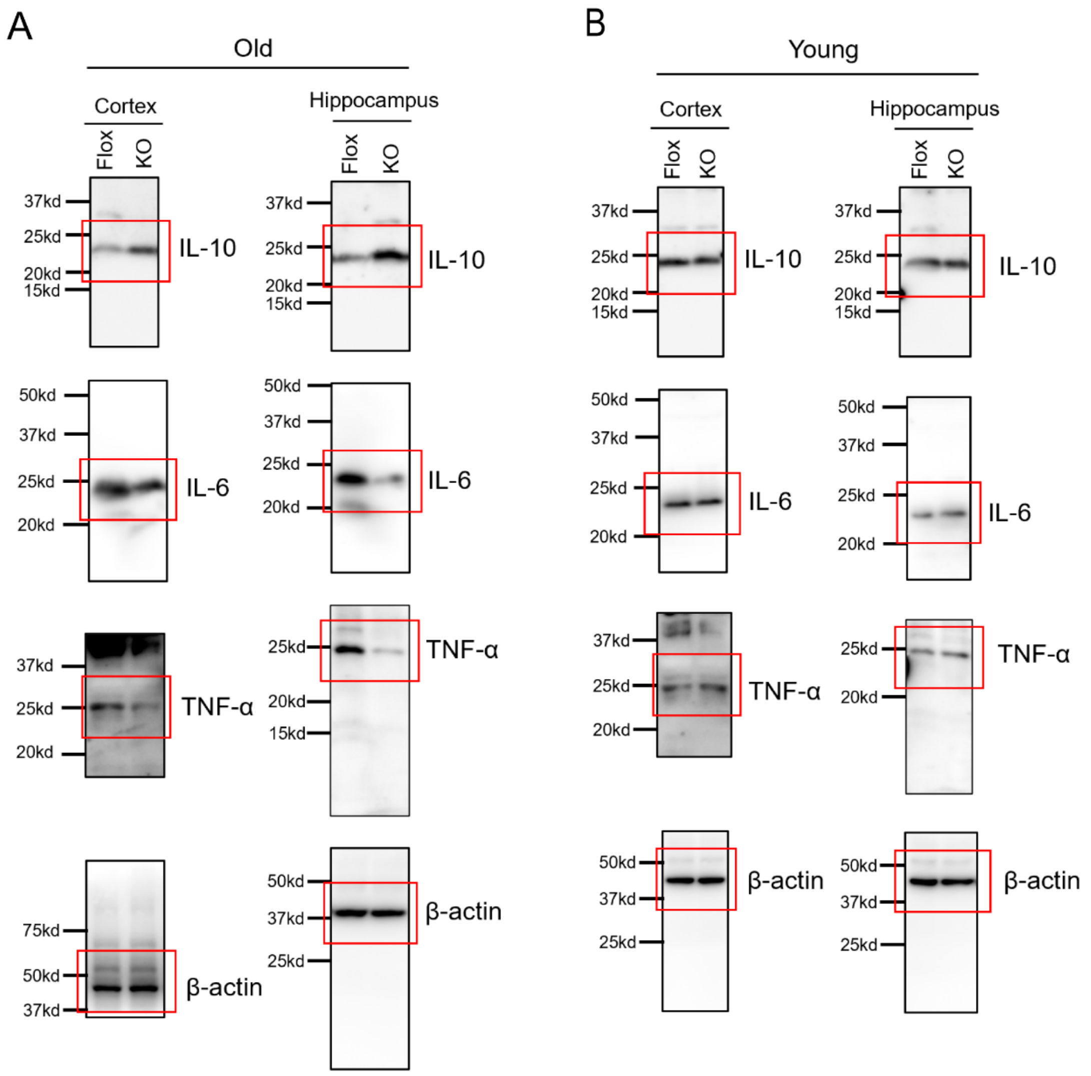

Supplementary Figure S3. Full-length, uncropped western blot images for cytokine analysis.

(A) Original, uncropped blots for IL-10, IL-6 and TNF-α from the cortex and hippocampus of aged mice, corresponding to the data presented in Figures 2G and 2H.

(B) Original, uncropped blots for IL-10, IL-6 and TNF-α from the cortex and hippocampus of young (5-month-old) mice, corresponding to the data presented in Supplementary Figures S2C and S2D.

Molecular weight markers (kd) are indicated. Relevant lanes used for final figures are outlined.
